# Ostracism via virtual chat room—Effects on basic needs, anger and pain

**DOI:** 10.1371/journal.pone.0184215

**Published:** 2017-09-06

**Authors:** Ana Paula Gonçalves Donate, Lucas Murrins Marques, Olivia Morgan Lapenta, Manish Kumar Asthana, David Amodio, Paulo Sérgio Boggio

**Affiliations:** 1 Social and Cognitive Neuroscience Laboratory, Center for Health and Biological Sciences, Mackenzie Presbyterian University, Sao Paulo, Brazil; 2 Developmental Disorders Program, Center for Health and Biological Sciences, Mackenzie Presbyterian University, Sao Paulo, Brazil; 3 Department of Humanities and Social Sciences, Indian Institute of Technology Kanpur, India; 4 Department of Psychology, New York University, New York City, New York, United States of America; 5 Department of Psychology, University of Amsterdam, Amsterdam, Netherlands; Purdue University, UNITED STATES

## Abstract

Ostracism is characterized by a social pain provoked by being excluded and ignored. In order to address the effects of social ostracism in virtual non-physical interactions, we developed a more realistic paradigm as an alternative to Cyberball and assessed its effects on participant’s expression of basic social needs, emotional experience and painful feeling. The chat room consisted of controlled social dialogue interactions between participants and two other (confederate) chat room partners. Exclusion was manipulated by varying the number of messages a participant received (15% and 33% in exclusion and inclusion, respectively). Analysis of participant (N = 54) responses revealed that exclusion induced a lower experience of basic-need states and greater anger, compared with included participants. In addition, excluded participants reported higher levels of two specific self-pain feelings, namely tortured and hurt. Our findings suggest that this procedure is effective in inducing social ostracism in a realistic and yet highly controlled experimental procedure.

## Introduction

Strong social bonds are necessary for human survival[1], and humans are acutely adept at recognizing and reacting to threats of exclusion associated with physical distance or signs of social avoidance [2]. Ostracism is a phenomenon characterized by a social pain provoked by being excluded and ignored by an individual or group[3–5]. In modern life, humans can experience social exclusion not only in traditional face-to-face interactions, but also in virtual environments. In order to address the effects of ostracism in virtual social interactions, we developed a new paradigm based on the internet chat room environment.

In prior research on ostracism in virtual environments, most studies have manipulated inclusion and exclusion using virtual ball tossing paradigms (Cyberball and Cyberbomb paradigms)[6]. Although inspired by a real-life experience of being excluded from a pick-up game of Frisbee in the park, the instantiation of this experience in games such as Cyberball are nevertheless limited in their ecological validity because people rarely, if ever, interact in such ways in real life. Instead, a much more common context for virtual social interaction (and exclusion) is the online chat room. Thus, the chat room context may offer a more powerful and realistic experimental paradigm for examining ostracism in a virtual environment that does not involve direct physical contact. In this approach, the participant can be led to believe that he or she is interacting with different individuals, when in fact they are being included or excluded in a predetermined manner by a computer. Given the significant real-life implications of ostracism in cultural, moral, legal, and social contexts, research using an ecologically-valid and socially-relevant approach is of great importance.

An early form of chat room interaction was used in at least two previous studies of ostracism [7,8]. These early approaches attempted to create ecologically-valid chatrooms. Gardner et al. [7] developed an online chat-room paradigm with three possible conditions: social acceptance, interpersonal rejection and collective rejection. Participants in the social acceptance condition received social agreement and positive feedback on their responses, whereas participants in both the interpersonal and collective rejection conditions received no interaction from confederates. Therefore, instead of only receiving an increased amount of interactions as in Cyberball paradigms [9], participants were socially included by receiving positive feedback, which represents a different form of rejection than exclusion, per se. In a different chat room procedure, used by Williams, Govan [8], ostracized participants were given a possible specific reason for their exclusion (e.g., because the participant doesn’t agree with a chat room partner’s opinion), which may provide the participant with a rationale for the exclusion and thus potentially limit the effect of ostracism.

A goal of the present research was to test a new, updated chat room paradigm that provides a cleaner and more realistic manipulation of social exclusion, in order to increase its construct validity with respect to previous work. In this new chat room paradigm, participants are able to freely choose virtual members to interact with and implicitly build bonds, without positive feedback or justification of interactions based on individuals’ self-interest. Thus participants are included *vs* excluded according to the amount of received interactions making it an alternative to Cyberball that is more realistic in some ways, however less realistic compared to other chat paradigms mentioned above for not allowing participants to engage in self-generated dialogue. The payoff of not allowing self-generated conversation is an adequate control of biases and a paradigm more amenable to experiments using neural or physiological measures.

To investigate the effects of our chat room paradigm, we tested the effects of ostracism on participants’ expression of basic social needs (belonging, self-esteem, control and meaningful existence). Furthermore, we assessed subjects’ emotional experiences after the chat room. We hypothesized that ostracism during the chat room interaction would decrease participants’ reported levels of basic needs. Also, we expected that excluded subjects would report higher ratings of anger, resentment and sadness as compared to the included group. In the current study, we focused on reflexive assessment by assessing emotional and basic needs scales immediately after the chatroom interaction. According to Williams [10], the direct reaction to ostracism is experienced in three progressive stages: (1) a reflexive stage, characterized by an immediate reaction without intentional thinking; (2) a reflective stage, characterized by deliberative thought, attributional inferences of motives, and the engagement of coping behaviours, and, when ostracism is prolonged, (3) a resignation stage, in which the ostracized individual shows exhaustion of all coping behaviors. It is worth mentioning that these forms are time related and it has been well documented that the reflexive reaction to ostracism is associated with an attenuation of belonging, control, self-esteem and meaningful existence.

## Materials and methods

### Participants

Sixty-six healthy female volunteers from a psychology undergraduate course volunteered to participate in this study. A female-only sample was used because women have been shown to be more sensitive than men to social exclusion [11] and social pain [12]. However, a meta-analysis published after this research was conducted suggests that such gender effects may be small or negligible [13]. Inclusion criteria were: (1) age between 18–34 years; (2) Brazilian native. Exclusion criteria were (1) regular medication intake; and (2) history of psychiatric or neurological treatment; (3) history of substance abuse or dependence; (4) use of central nervous system-effective medication; (5) high scores on depressive scales (please see scale subheading). Twelve participants were excluded from the data analysis: three due to high scores on anxiety and depression scales (greater than 3 SDs), seven due to expressed suspicion of the chatroom procedure (although they reported high pain scores when in the exclusion group), and two due to questionnaire completion errors. Thus, the final sample included 54 subjects (47 right-handed, mean age = 22.38 ± 3.93). This study was conducted in accordance to the ethical standards of the Declaration of Helsinki and was approved by the Institutional Ethics Committee at Mackenzie Presbyterian University, Brazil, and by the National Research Ethic Committee (SISNEP, Brazil; CAAE n° 03733612.2.0000.0084). All participants gave written informed consent and received extra credits in their course for their participation.

### Procedure

Participants who met inclusion criteria were randomly assigned to one of two experimental chat room conditions: (1) Inclusion; or (2) Exclusion (see chat room experimental condition section). Before responding to the questionnaires, participants met the other two (confederate) chat room partners, and we checked that none of the participants knew each other previously. After greeting participants, the experimenter took pictures of the participants and the two confederates, each posing with neutral facial expressions in front of a white background. Next, participants were seated in separate rooms and then the real participant completed questionnaires and performed the chatroom task. To control possible environmental interference, the chat room was conducted in a silent room in which participants were seated comfortably at 60 cm from the monitor. Both unknown individuals were, in reality, laboratory members, who voluntarily gave their facial pictures to the experimenter. At the end of the experimental protocol, the real purpose of the study was revealed to the participant and it was explained that all interactions were pre-programed.

#### Scales

Before the experimental task, all participants responded to demographic questionnaires (age, medication, health history, drug use) and the following scales: Beck Depression Inventory (BDI) [14], Beck Anxiety Inventory (BAI) [15], Social Ability Inventory (SAI) [16], Rosenberg scale of self-esteem [17], Social Desirability [18] and Resilience Evaluation Scale (RES) [19]. Immediately after the experimental task, participants completed scales regarding: emotional experience, basic needs, and self-pain. Specifically, subjects reported how much they experienced each of a list of emotions, using a visual analogue scale (VAS) [20] with values from 1 (not at all) to 7 (extremely), for the words Anger, Sadness, Happiness and Resentment. We focused on these specific emotion variables that are known to be most sensitive to the impact of ostracism on the self [13]. Participants were instructed to report their feelings after the chat with regard to each of these words.

Next, participants rated their endorsement of basic psychological needs, using the Need Threat Scale (NTS) [21], in which twenty sentences are presented and participants answer a VAS ranging from 1 (totally disagree) to 7 (totally agree) (4). These sentences are grouped in four general basic needs categories: belonging (e.g., “during the chat-room I felt alone”), control (e.g., “during the chat-room I felt in control”), self-esteem (e.g., “during the chat-room I felt insecure”), and meaningful existence (e.g., “during the chat-room I felt that my presence was meaningful”). Concerning self-pain assessment, participants were instructed to answer a subjective scale [22] in which they reported their experience of pain-related words (torture, hurt, and sore) from 1 (totally disagree) to 7 (totally agree). Finally, subject reported: (i) how much they believed to be in a real chat-room conversation and the percentage of questions they perceived to have received from the other participants (from 0 to 100%); (ii) how painful it was to participate in the chat interaction (from 1 to 10). These scales were chosen based on previous studies showing that social pain induced by experimental condition is related to increased negative sensation as well as self-pain evaluation [21, 23].

#### Chat room experimental condition

The chat room consisted of 210 social dialogue interactions presented using E-prime 2.0 (E-prime^®^, http://www.pstnet.com/eprime.cfm) via a 15’ monitor (Lenovo, 4434HE1). The chat is organized in such a manner that each participant in each and every round has a turn to propose a question to another participant. On each round, participants were free to choose who would answer their question.

**Fig 1:**
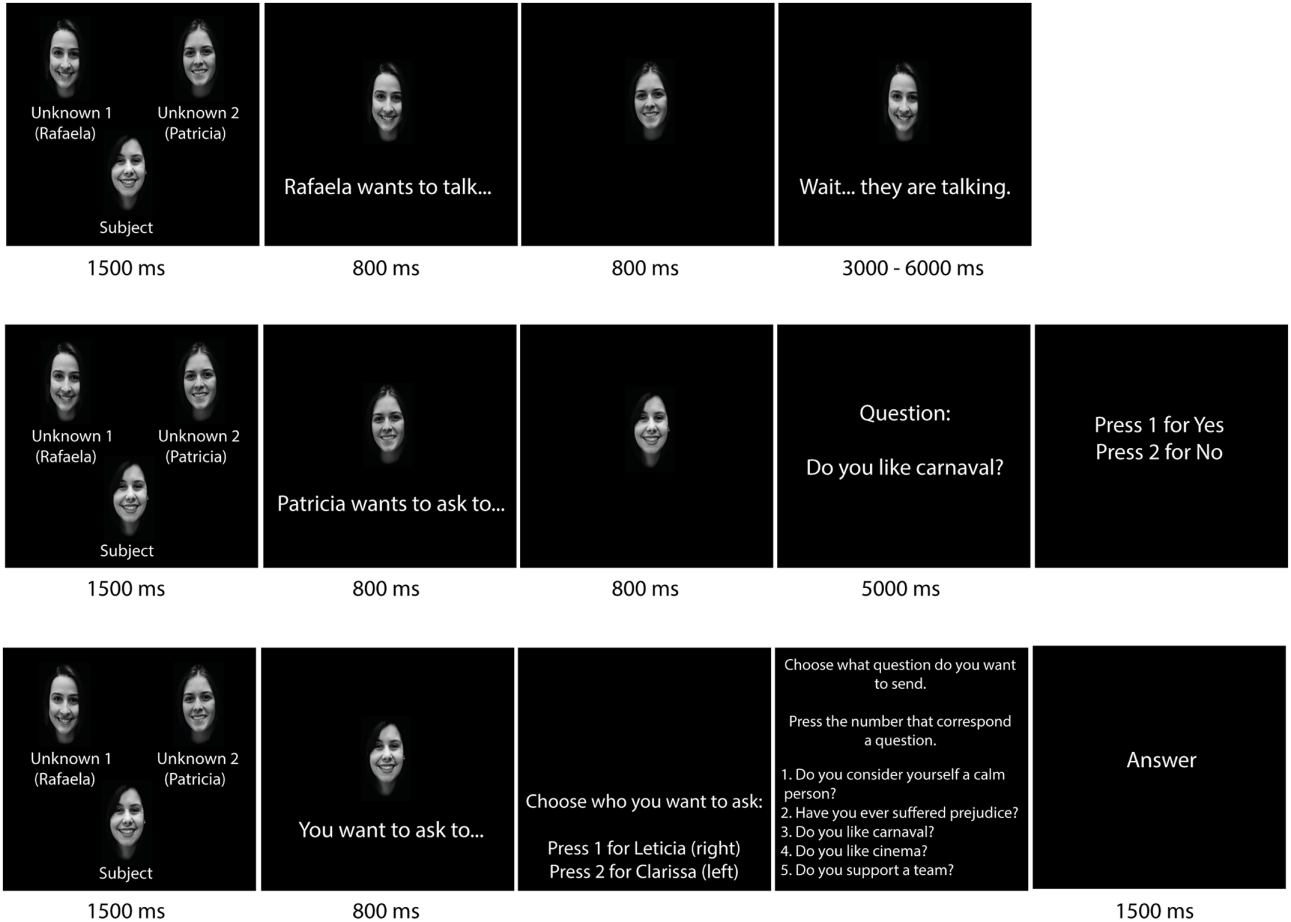
Experimental design of chat room. a) Confederate participants talking between themselves; b) Participant receiving questions from one of the fictitious participants c) The participant chooses one of the fictitious participants to direct a question of their choice. The individuals in this manuscript has given written informed consent (as outlined in PLOS consent form) to publish these case details.

Accordingly, three possible situations could be experienced by the real participant on a given trial (please see Fig 1): (a) the participants view the confederates chatting with each other; (b) the participant is solicited by a confederate to respond; (c) the participant solicits a confederate to respond. In the first situation, the participant simply observes one of the confederates choosing the other one to answer a question, and then, the participant received the following sentence as feedback in the screen “Waiting… The other participants are chatting”. In the second situation, one of the confederates delivers a question to the participant, who in turn selects an answer. Possible answers to all questions were either "yes" or "no" (pressing button “1”–“yes” or “2”–“no”). In the third situation, the participant selects and delivers a question to one of the confederates.

At the beginning of each trial, pictures (3x2cm size) of the confederates were presented in the screen, and the participant chose whom to interact with by pressing button “1” or “2” (“confederate 1” or “confederate 2” respectively). After that, the participant chose one question to deliver out of five options (ex.: “are you vegetarian?”, “do you practice sports?”, “do you have brothers?”, “have you ever travelled by boat?”, “do you enjoy watching TV series?”), using numeric keys from “1 to 5” on keyboard. All the possible questions were previously programmed, and all participants were presented with the same questions, sequence, and intervals. Lastly, the confederate response was presented to the participant.

Participants in both conditions were able to choose a question and a person to direct it to in a total of 70 trials. In the inclusion condition, participants received the same number of questions from each of the other chat room partners; thus their responses were solicited by others on 33% of rounds. In the exclusion condition, participant received fewer questions than others, being asked to respond on only 15% of the rounds. Importantly, contrary to classic Cyberball paradigms [9], in which the participant under the exclusion condition receive few inclusion trials only at the beginning of the experiment, the exclusion condition is characterized for receiving a lower percentage of inclusion trials that are randomly presented throughout the experiment. Thus, compared to previous Cyberball paradigms, in which the participants in the exclusion condition were completely excluded by confederates [6], the present paradigm involves partial exclusion, with interaction on 15% of the rounds. This form of partial exclusion may be more subtle than full exclusion, yet it may resemble a wider range of exclusion situations.

Furthermore, it is worth mentioning that the person who answered one question did not necessarily deliver the following question. Specifically, the selection of who initiates each interaction was randomly determined by the computer in such a way that all participants delivered a question on a total of 70 trials. The entire task lasted approximately 50 minutes.

## Results

### Pre-task scales

Student’s t-test revealed no statistically significant differences between conditions on any pre-task measures (see Table 1). Furthermore, we calculated the Cronbach’s alpha for each scale: BDI (α = .75); BAI (α = .82); Rosenberg Self-esteem (α = .81); Social Desirability (α = .66); SAI (α = .76); RES (α = .85).

**Table 1:** Mean (standard deviation) and statistical analysis of baseline questionnaires before the virtual chat-room experience (inclusion, exclusion) in virtual chat-room paradigm.

	Exclusion (28)	Inclusion (26)	*t*-value	*p*-value
**BDI**	6.54 ± 3.83	7.35 ± 3.44	-0.81	.42
**BAI**	7.14 ± 6.00	8.54 ± 5.17	-0.91	.37
**Rosenberg**	31.54 ± 3.70	31.54 ± 4.65	-0.002	1.00
**Social Desirability**	9.29 ± 2.76	9.29 ± 3.49	-0.0005	1.00
**SAI**	96.29 ± 14.63	96.77 ± 14.66	-0.12	.90
**RES**	3.65 ± 0.43	3.59 ± 0.43	0.56	.58

### Exclusion effects on emotional experience

**Fig 2:**
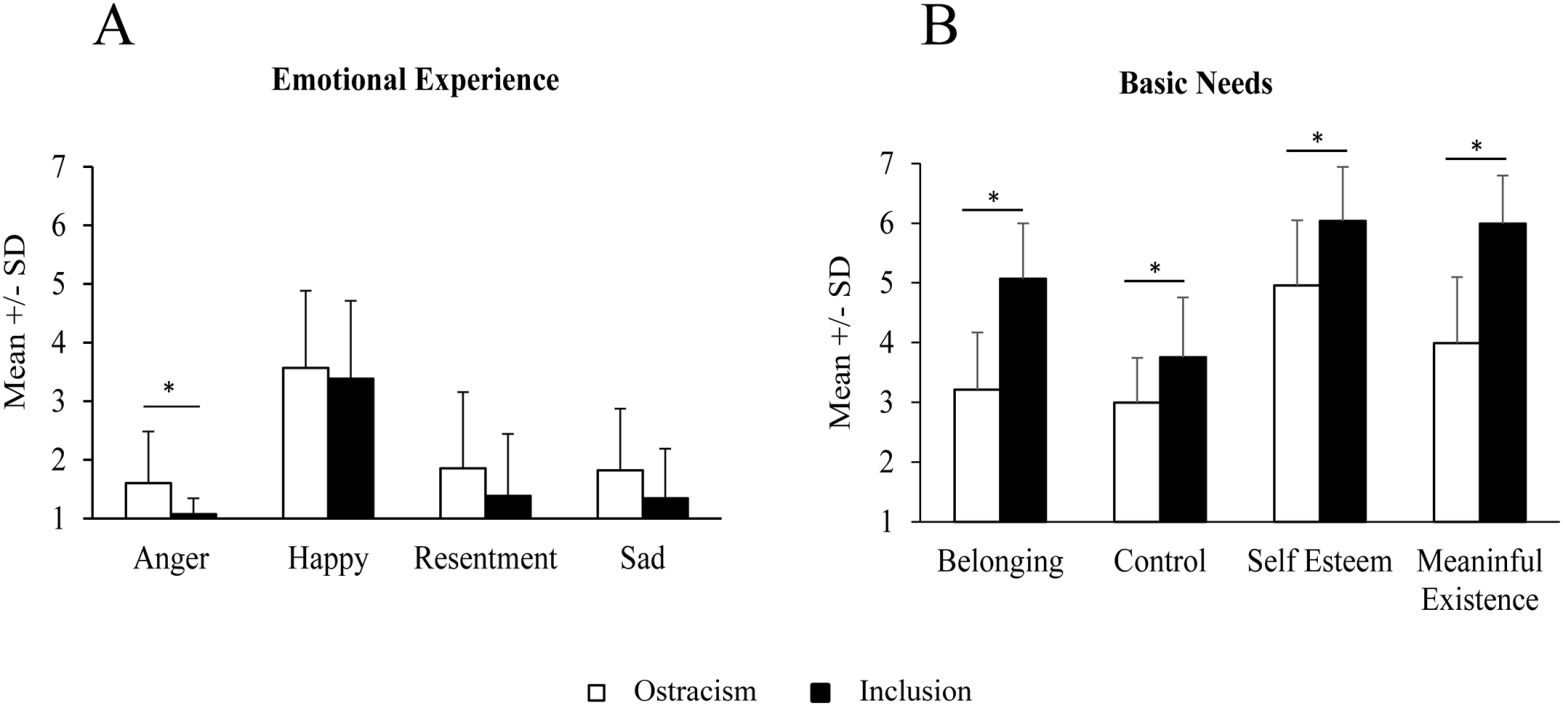
A: Impact of exclusion or inclusion over emotional experience. B: Impact of exclusion or inclusion over basic needs. Values are described as mean +/- Standard Deviation. * p< .05.

The effect of condition on emotional responses was tested, using univariate analysis of variance (ANOVA), for each of the evaluated emotions. No differences emerged for resentment (F[1,52] = 2.13; *p =* .15; η_p_^2^ = .04), sadness (F [1,52] = 3.30; *p =* .08; η_p_^2^ = .06), or happiness (F[1,52] = 0.27; *p =* .61; η_p_^2^ = .005). However, we observed a significant effect for anger (F[1,52] = 8.75; *p =* .005; η_p_^2^ = .14). Specifically, excluded participants (M = 1.61, SD = 0.88) reported greater anger after the chat room experience than included participants (M = 1.08, SD = 0.27; Fig 2). The analysis of the effects of ostracism on the basic needs between experimental groups, considering all participants (n = 66), resulted in similar and significant effects. We found a main effect of group (F[1,64] = 37.59; p< .000001; η _p_^2^ = .37), subscales (F[3,192] = 76.04; p< .000001; η _p_^2^ = .54) and the interaction between groups and subscales (F[3,192] = 7.48; p< .0001; η _p_^2^ = .10). Pairwise comparison revealed significant effects for belonging (inclusion M = 5.02 SD = 0.87; exclusion M = 3.39 SD = 1.08; p< .001); control (inclusion M = 3.70 SD = 1.01; exclusion M = 3.04 SD = 1.01; p< .010); self-esteem (inclusion M = 5.88 SD = 1.30; exclusion M = 5.00 SD = 1.21; p< .006); meaningful of existence (inclusion M = 5.82 SD = 1.00; exclusion M = 4.00 SD = 1.18; p< .001).

### Exclusion effects on basic needs

The NTS was scored as in Van Beest and Williams [9], such that higher scores reflect greater satisfaction of the given need (i.e., with reverse scoring as appropriate). Composite scores for each need type were created by averaging scores on the five items representing each need. The scores were analysed using multivariate analysis of variance (MANOVA), using the experimental condition (inclusion or ostracism) and each of the basic needs as a factor. When significant, pairwise comparisons were conducted between groups for each basic needs categories.

A MANOVA on all four subscales revealed a significant effect for group (F[1,52] = 56.18; p< .000001; η _p_^2^ = .52), subscales (F[1,52] = 84.80; p< .000001; η _p_^2^ = .62) and the interaction between groups and subscales (F[3,156] = 8.637; p< .000024; η _p_^2^ = .14 with p). Consistent with prediction, pairwise comparison revealed lower scores in the exclusion than inclusion conditions for belonging (p < .001), self-esteem (p < .001), meaningful existence (p < .001) and control (p = .003), as shown in Fig 2.

Furthermore, we calculated the Cronbach’s alpha for each NTS aspect: Belonging (α = .65); NTS Control (α = .50); NTS Self-esteem (α = .77); NTS Meaningful existence (α = .86).

### Exclusion effects on self-reported social pain and painful experience

A univariate ANOVA was performed on the average of the hurt-related emotional words between conditions. This analysis revealed a significant difference between groups (F[1,52] = 5.87; p = .02; η_p_^2^ = .10), such that excluded participants reported higher levels on pain-related words as compared to the included ones (Table 2). A Univariate ANOVA was also performed on the average measure of painful experience due to their participation in the chat interaction. The results demonstrated that participants in the excluded condition reported significantly higher levels of painful experience than the included participants (F[1,52] = 7.98; p = .007; η_p_^2^ = .13).

**Table 2:** Mean of self-report ratings after the virtual chat-room experience for each group and statistical results of comparison between groups (inclusion, exclusion).

	Exclusion (28)	Inclusion (26)	F-value	P-value	η_p_^2^
**Received Messages**	16.61 ± 5.99	35.00 ± 11.74	53.72	< .0001	.51
**Believe**	81.43 ± 21.34	88.46 ± 17.88	1.71	.20	.03
**Painful Experience**	2.29 ± 1.54	1.26 ± 1.04	7.98	.007	.13
**Pain-related words**	1.56 ± 0.86	1.12 ± 0.34	5.87	.02	.10

### Manipulation check

Univariate ANOVA was used to test group effects on self-report ratings of virtual chat-room experiences. The results demonstrated that participants in the excluded condition felt that they received significantly less percentage of messages than participants on the inclusion group,. As can be seen in Table 2, the perception of how many messages they received during the chat is similar to the real percentage of received messages (exclusion group: 15%; inclusion group: 33%). Finally, groups did not differ in the extent to which they believed the chat room interactions were real (see Table 2).

## Discussion

Online chatrooms constitute an increasingly common context for social interaction and, in many cases, exclusion. The goal of the present research was twofold: to develop a new paradigm for examining exclusion in the online chatroom context that is realistic while also permitting experimental control, and to examine the effect of exclusion on reported social emotion, social needs, and experienced social pain within this chatroom context. Overall, the chatroom paradigm proved effective. We found that (i) participants believed to be interacting in a real chat room, evidenced by the high believability scores and accurate perceptions of chat room interactions among participants in both groups; (ii) the exclusion chat room condition elicited the effects of exclusion on basic needs, such that ostracized participants felt that their basic needs were threatened, with lower scores concerning self-esteem, control, meaningful existence, and belonging in comparison to included participants; (iii) exclusion condition evoked anger after the chat experience; (iv) participating in the chat was reported as more painful for the ostracized group compared to the included group and furthermore, ostracized participants had higher scores in self-pain assessment.

Thus, we successfully developed a highly-controlled virtual interaction using a context of common interactions observed in virtual relationships. It could be argued that participants might have guessed that the chat room was not real. Notwithstanding, participants reported to be around 80% sure that the interaction was real. The included group reported a slightly higher degree of believability; specifically 88% versus 81% of the ostracized group. In general, previous studies usually mention the total number of suspicious participants without considering group and strength of belief (e.g., [24, 25]). We opted for a more transparent evaluation because differences in suspicion could reflect an attempt by ostracized participants to find an explanation for their exclusion other that their own behaviour, perhaps as a defensive response to the negative situation [4]. According to the Need Threat Model, the reflective stage allows people who are ostracized to assign reasons for ostracizing situations, which could result in suspicion of the paradigm. However, excluded and included groups did not differ significantly in their reported suspicion. Finally, previous studies claim that even when a participant knows they are playing against a pre-programed computer, exclusion still promotes feelings of exclusion [26]. Thus, even if a participant were suspicious, he or she would still likely experience a genuine exclusion response.

Our chatroom procedure differed in some notable ways from previous ostracism paradigms. For instance, unlike Cyberball or Cyberbomb, in which participants do not know each other before and after each interaction and no personal information is shared between participants [3, 4], our virtual chat room interaction commences following a brief encounter with other participants and allows the participant to propose questions (previously elaborated) for the other participants. We also changed some details that made our chat different from previous paradigms using virtual conversations. For example, in Williams and Govan [8], the ostracized participant is given a reason for their exclusion. For example, they are clearly shown to be a member of an “outgroup” (i.e., different school or different point of view).

Our chatroom procedure also differs in important ways from an early paradigm used by Gardner, Pickett and Brewer [7]. In this work, groups were instructed to participate in an “indirect impression formation” study in which participants ruminate on the impression they convey to the other confederates. In our chat-room paradigm, participants were only instructed to join in a chat-room paradigm, without being led to direct their attention to any specific issue (e.g., the impression they made on others). Moreover, unlike Gardner’s paradigm, in which participants were not introduced prior to the task, in the present study participants were introduced to each other, and we ensured that none were previously known to the participant. Thus, our procedure enhanced the believability of the interaction while also controlling for participants’ perceptions and knowledge of the other chatroom participants. Also, participants in Gardner, Pickett and Brewer [7] inclusion condition received affirmational feedback (e.g., social agreement and acceptance responses), whereas inclusion and exclusion in the present work differed only in terms of the relative amount of times the interaction partners solicited their response as opposed to the other partner. Finally, our participants, in turn, voluntarily chose the virtual member they wanted to interact with in each round, and the exclusion was conveyed through receiving fewer questions. To make participants aware of their exclusion (i.e., when they are not chosen for an interaction), the following message appeared on the screen “wait a moment, the other participants are chatting,” but since all questions are pre-programed, they are not given a reason for exclusion. Thus, having no apparent reason to be ostracized could increase the exclusion impact because the individual keeps seeking an explanation in order to enable adjustments to fit the group and avoid the persistence of the ostracism [4]. This design is also similar to some real situations such as preliminary interactions with unfamiliar people on the internet. However, we pre-determined a list of questions that participants could choose and restricted the responses to yes/no answer. In this way, we were able to control the nature of questions that participants from both groups received. One could see the forced choice aspect of the questions within the procedure as a limitation. Indeed, such an approach limits interactions, contrasting most virtual chat environments. However, by avoiding free choice of chat topics, it is possible to control and maintain chat content equally distributed among participants, allowing direct observation of the effects of being included versus excluded. Furthermore, this design allows **to** manipulate the content of a conversation and evaluate if specific topics (e.g., about other people, or ideas, or objects) mostly impact the basic needs and emotions of the ostracized subjects. Moreover, it is advantageous for studies coupling behavioral and physiological measures that require more controlled scenarios such as neuroimaging, electroencephalography and psychophysiological data recording.

With all this, our chat room paradigm might be useful for neurophysiological assessments during and following ostracism. An interesting approach would be to study the effects of transcranial direct current stimulation (tDCS) over Ventrolateral Prefrontal Cortex (VLPFC) and Dorsolateral Prefrontal Cortex (DLPFC) during virtual social interactions. Based on Riva and colleagues’ [27] findings of effective attenuation of ostracism’s effects on basic needs, and neuroimaging findings of Nishiyama, Okamoto [28] and Onoda, Okamoto [29] on the correlations of DLPFC activation with lower post ostracism effects, we believe that both these frontal areas could differently modulate the consequences of ostracism and thus help participants suffering from ostracism to react in a more survival-oriented way by boosting and speeding up the second stage of the Temporal Need Threat Model of Ostracism [10]–coping. This could potentially allow ostracized individuals to have increased scores on self-esteem, meaningful existence, belonging and control to the level of participants included in the chat room. Alternatively, active tDCS could attenuate the feeling of being hurt by exclusion. The possibility to increase basic needs levels, while increasing arousal, may benefit patients suffering from depression and anxiety, which represents an impairment in mood and basic needs control [30, 31]. Lastly, integrating neuromodulation with different montages combined with neuroimaging techniques could compare the effects over left and right hemisphere and contribute to the discussion on functional asymmetry and in the overlap between social and physical pain discussions.

The efficacy of this chat-room can be seen in its effects on basic needs. We found lower scores on belonging, self-esteem, control and meaningful existence in the ostracized participants as compared to the included ones. Our data are aligned with previous studies showing decreased ratings on basic needs for excluded groups compared with included groups [4, 8, 9]. The effects on basic needs produced by being included or ostracized might be due to our social nature and basic motivation to establish social connections. Being ostracized might be seen and felt as being banned from a group. In this sense, it is interesting to see that excluded participants reported lower levels of belonging and meaningful existence. Self-esteem was also affected, which might have led to changes in self-concept in response to negative social feedback. Importantly, these effects were observed in a scenario of partial exclusion, revealing that even milder forms of ostracism are threatening. These effects are the negative aspects of being ostracized and might be directly connected (under long term exposure conditions) to pathologies such as depression, anxiety, and phobic disorders [11, 32–36]. Additionally, in extreme cases of cyber ostracism and bullying, the negative effects on basic needs might result in dramatic consequences such as suicide or crimes in schools [37].

We also found effects of this chat-room procedure on anger, but not sadness, resentment, or happiness. This finding is consistent with Greene [38], who proposed that friendship might be seen as a cooperation device that evolved over the course of human history. A failure, rupture, or avoidance during the establishment of a new relationship (as in the exclusion condition) might induce moral emotions such as anger (a primary one) and resentment (a more complex one). By contrast, enhanced happiness is usually observed when one is included in a social interaction, though we did not observe differences in happiness between conditions. The effect of exclusion on anger corroborates previous findings on behavioural responses to social exclusion [39]. However, the lack of effects in other emotional experiences might be due to the fact that we evaluated these emotions only at the end of the chat-room session and, hence, we do not know whether these emotions might have been engaged and then regulated by excluded participants. Moreover, to understand how ostracized individuals responded, we must also consider the ratings on “painful words” which relate to reflexive responses in the ostracized group. Ostracized participants reported higher levels of pain-related words in comparison to included participants.

Given the potentially important applications of this paradigm, some limitations should be raised in order to enable improvements. First, we tested only females, and so we cannot claim that our findings will also pertain to males. However, given a recent meta-analysis suggesting no difference between females’ and males’ reactions to exclusion [13], we expect that our results would generalize. Further research is necessary to examine effects in the general population. In addition, although the chat room was designed to resemble key elements of a real-life online interaction, the procedure was highly-controlled, with some constraints that nevertheless limit the extrapolation of our findings to naturalistic situations to an extent. Another limitation is the fact that we only evaluated participants immediately after the chat experience, and therefore focused only on reflexive stage responses. In future research, it would be interesting to measure emotional experience and basic needs at different time points during the task to examine both reflexive and reflective responses.

## Supporting information

S1 Table Raw data. This table contains the raw data of all scales and questionnaires used in this experiment.
(PDF)
