# Supplementary material for: Ostracism via virtual chat room—Effects on basic needs, anger and pain
Source: PLoS One. 2017 Sep 6;12(9):e0184215. doi: 10.1371/journal.pone.0184215 (PMC5587273; doi:10.1371/journal.pone.0184215)
Supplement: S1 Table — This table contains the raw data of all scales and questionnaires used in this experiment. (PDF) [file pone.0184215.s001.pdf]

ID	Age	Group	Handedness	BDI	BAI	Rosenberg
2	20	Exclusão	right	4	5	31
4	22	Exclusão	right	3	1	31
6	19	Exclusão	right	8	4	26
9	22	Exclusão	right	5	3	27
12	18	Exclusão	right	6	11	32
14	22	Exclusão	right	5	3	28
19	21	Exclusão	right	4	7	29
20	30	Exclusão	right	9	9	34
25	18	Exclusão	right	3	3	31
26	21	Exclusão	left	7	12	30
27	18	Exclusão	right	13	10	35
28	20	Exclusão	right	7	3	27
29	20	Exclusão	right	4	1	34
30	19	Exclusão	right	7	26	33
33	19	Exclusão	left	5	15	35
36	21	Exclusão	right	7	9	31
38	31	Exclusão	right	12	8	31
40	23	Exclusão	right	16	19	28
43	20	Exclusão	right	2	3	31
44	20	Exclusão	left	5	10	26
45	18	Exclusão	right	9	14	28
47	32	Exclusão	right	4	5	34
48	23	Exclusão	right	3	3	39
49	23	Exclusão	right	16	8	28
51	19	Exclusão	right	1	2	39
52	21	Exclusão	right	7	3	32
53	23	Exclusão	right	4	1	36
54	23	Exclusão	right	7	2	37
1	21	Inclusão	right	9	6	26
3	26	Inclusão	right	9	2	33
5	25	Inclusão	right	7	3	31
7	24	Inclusão	right	10	14	28
8	25	Inclusão	right	8	9	37
10	24	Inclusão	right	9	10	30
11	24	Inclusão	right	1	1	38
13	20	Inclusão	right	7	11	31
15	20	Inclusão	right	4	5	32
16	33	Inclusão	right	4	3	38
17	23	Inclusão	right	9	8	26
18	22	Inclusão	right	5	8	30
21	20	Inclusão	right	4	6	33
22	30	Inclusão	right	4	3	37
23	21	Inclusão	right	0	1	35
24	19	Inclusão	right	12	10	20
31	20	Inclusão	left	14	15	27

32	20	Inclusão	right	7	2	26
34	20	Inclusão	right	4	10	37
35	25	Inclusão	left	13	9	26
37	34	Inclusão	right	10	14	38
39	19	Inclusão	right	8	14	33
41	21	Inclusão	right	8	16	31
42	19	Inclusão	right	11	13	31
46	26	Inclusão	left	8	20	31
50	22	Inclusão	left	6	9	35

Social Desira	SAI	RES	Anger	Happiness	Resentment	Sadness
11	112	4	1	2	1	1
9	92	3	1	2	1	1
6	104	3	4	3	1	2
5	85	4	1	4	2	1
9	113	4	3	2	6	4
8	89	4	1	4	1	1
9	82	3	1	4	2	1
10	85	4	1	4	1	1
8	83	3	3	4	3	2
6	81	4	2	2	2	3
15	106	4	1	3	3	2
10	91	3	1	2	1	4
11	105	3	1	5	1	1
5	82	3	1	4	3	4
6	123	4	2	6	1	1
9	95	3	2	2	2	2
9	81	3	1	4	5	1
8	83	4	3	4	3	4
11	108	3	1	4	1	1
15	106	4	1	5	1	2
10	81	4	1	5	1	1
11	105	4	2	4	1	2
9	98	4	2	2	3	2
13	78	4	1	1	1	1
12	112	4	1	5	1	1
4	80	3	3	3	2	2
11	132	4	1	4	1	1
10	104	4	2	6	1	2
9	91	3	1	1	6	4
8	121	4	1	4	1	1
9	96	3	1	4	1	1
9	82	4	1	2	1	1
12	109	4	1	5	1	1
8	110	3	1	3	1	1
8	93	4	1	1	1	1
7	78	3	1	3	1	1
9	110	3	1	5	1	1
1	103	3	1	5	1	1
6	58	4	1	4	2	2
8	102	4	1	4	1	1
13	92	4	1	5	1	1
11	99	4	1	4	1	1
17	96	4	1	4	1	1
12	71	3	1	4	1	1
6	104	3	1	2	2	2

10	103	4	1	1	1	1
13	111	4	1	1	1	1
10	68	3	2	4	3	2
9	101	4	1	3	1	1
11	103	3	2	5	1	1
5	108	4	1	4	1	1
3	105	3	1	4	2	4
14	101	4	1	3	1	1
13	101	4	1	3	1	1

Belonging	Control	Self-esteem	Meaningful e	Received me	Painful exper	Believe
11	11	15	17	15	6	70
10	7	18	18	25	2	50
11	13	24	25	15	2	80
19	18	19	21	10	2	100
10	7	20	12	10	6	100
17	17	25	24	20	5	100
18	14	30	20	20	3	100
22	16	30	26	20	1	80
13	14	22	11	10	3	100
14	14	23	22	20	3	100
15	17	30	21	25	2	100
15	17	30	21	20	3	100
16	25	32	23	15	1	50
8	10	18	13	15	4	100
23	19	30	22	33	1	100
17	18	29	19	10	2	65
18	16	18	20	9	4	45
16	18	20	22	20	2	80
22	13	28	25	20	1	90
25	13	35	30	20	1	100
22	16	25	19	10	1	50
17	19	27	32	13	1	40
5	18	23	16	20	3	100
20	14	16	13	10	1	50
14	14	29	19	10	1	70
17	15	21	18	20	1	70
15	12	31	7	10	1	100
20	14	26	23	20	1	90
17	23	25	22	85	1	100
22	11	29	32	30	1	100
32	15	35	30	45	1	100
20	16	21	21	20	6	95
24	27	34	33	33	1	85
27	24	26	30	33	1	50
27	11	29	35	33	3	50
18	13	28	28	50	1	100
28	18	31	33	33	1	80
16	15	23	25	32	1	70
25	19	22	29	33	1	100
22	11	34	25	33	1	100
32	28	31	32	33	1	100
23	17	31	28	33	1	90
22	24	25	23	33	1	100
24	22	28	29	33	1	100
24	21	26	28	40	1	100

23	17	35	35	33	1	100
31	18	35	33	20	1	100
30	16	35	33	30	1	100
28	26	34	33	33	1	80
26	18	35	30	33	1	80
33	16	31	33	33	1	80
28	16	33	31	33	1	100
28	26	34	33	33	1	100
29	20	35	35	30	1	40

Pain-related words
3
1
2
1
3
1
1
1
1
2
1
1
1
1
1
2
2
4
1
1
1
1
3
1
1
1
2
2
1
1
1
2
1
2
2
1
1
1
1
1
1
1
1
1
1

1
1
1
1
1
1
1
1
1
